# Experimental study on the effect of microwave heating power on the drying behavior of onion slices

**DOI:** 10.1016/j.mex.2025.103479

**Published:** 2025-07-02

**Authors:** G Srinivas, G Bhanu Radhika, BVS Praveen

**Affiliations:** a Department of Chemical Engineering, B V Raju Institute of Technology, Narsapur, Medak 502313, Telangana, India; b Department of Chemical Engineering, Chaitanya Bharathi Institute of Technology (CBIT), Hyderabad 500075, Telangana, India

**Keywords:** Microwave drying, Onion slice, Dehydration process, Instantaneous moisture content

## Abstract

Microwave drying has become a highly effective and energy-efficient technique for the processing of agricultural products, mainly onion slices. This method proves to be advantageous over traditional methods of drying like sun drying and hot-air drying, which are often accompanied by longer periods of drying time and quality loss in terms of color, texture, and nutrient content. Microwave drying allows moisture removal through volumetric heating and thus saves energy while preserving essential quality attributes. This study examines the effects of microwave power levels ranging from 200 W to 1000 W on the drying behavior and quality attributes of onion slices of different thicknesses. The results showed that higher microwave power levels enhance drying rates but may cause undesirable quality degradation, such as color changes and texture softening. Mathematical models were developed to predict instantaneous moisture content and drying kinetics, which are crucial for optimizing drying processes. The study highlights the need for microwave power balance, drying time, and slice thickness to preserve the quality of the product but increase the efficiency of drying. Findings can contribute to developing optimized microwave drying protocols to achieve improvements in energy efficiency, quality of the products, and sustainability in agricultural processing.➢Higher power levels increase efficiency but may degrade product attributes. ➢Texture analysis was conducted on onion slices dried at different microwave powers. ➢Color analysis of onion slices dried at different microwave power levels.

## Graphical abstract

This graph shows the relationship between microwave power (W) and average L values, which represent the lightness of onion slices after drying. As the microwave power increases from 200 W to 1000 W, the average L values decrease significantly, indicating a reduction in the lightness of the onion slices. The trend suggests that higher microwave power levels cause greater color degradation, likely due to accelerated drying rates and potential overheating. This highlights the importance of optimizing microwave power to balance drying efficiency with quality retention in terms of color.

**Figure:**
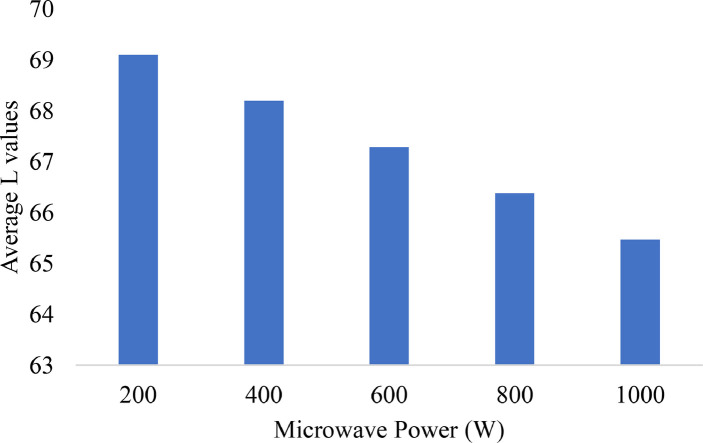

**Specifications table**

**Table:** 

**Subject area**	Agricultural and Biological Sciences
**More specific subject area**	*Drying of sliced onions*
**Name of your method**	*Microwave drying with variable power levels from 200W to 1000W*
**Name and reference of original method**	*Feng, H., Yin, Y., & Tang, J.* [1]*. Microwave drying of food and agricultural materials: Basics and heat and mass transfer modeling. Food Engineering Reviews, 4, 89–106.*https://doi.org/10.1007/s12393-012-9048-x
**Resource availability**	

## Background

Microwave drying has become a highly effective and energy-efficient method for the processing of agricultural products, particularly onion segments. Microwave drying has great benefits over more traditional techniques like sun drying and hot-air drying, which can produce long drying durations and noticeable quality loss. Because of unequal or prolonged heat exposure, traditional techniques often cause breakdown in color, texture, and nutritional content. By use of volumetric heating, on the other hand, microwave drying enables quick and consistent moisture elimination from inside the product. This is a possible substitute for large-scale agricultural processing since it not only lowers general drying time but also helps preserve important quality criteria. The objective of this work is to investigate the impacts on the drying kinetics and qualitative attributes of onion slices with different thicknesses of several microwave power levels, ranging from 200 W to 1000 W. The results show that although greater power levels hasten the drying process, they may also cause undesired quality alterations including color modification and texture softening.

Mathematical models were used to forecast the instantaneous moisture content and drying behavior of onion slices so that one may better grasp and maximize the drying process. These models are vital instruments for adjusting the drying conditions to guarantee both effectiveness and product quality. To improve energy economy and preserve the integrity of the last product, the study emphasizes the need of harmonizing microwave power, drying time, and sample thickness.

The results of this work provide insightful analysis of the evolution of ideal microwave drying techniques, therefore improving sustainability and product quality in the agricultural processing sector.

## Method details

### Introduction

Microwave drying has emerged as a highly efficient method for processing agricultural products, particularly onion slices, where preserving quality and minimizing energy consumption are critical goals [2]. Traditional drying methodologies include sun-drying, convective drying and hot-air-drying, limited to long-lasting times and possibilities of quality changes that may harm the color aspect, texture aspects and nutritional parameters of the raw materials [[3], [4], [5]]. On the other hand, microwave drying has volumetric heating, which rapidly removes moisture, significantly reduces drying time, and retains quality attributes more effectively [[1], [6],^7^].

There are many studies that point out the advantages of microwave drying compared to traditional drying methods. For example, Arslan and Ozcan [4] reported that onion slices dried by microwaves possessed better color and nutritional quality compared to sun- and oven-dried samples [8,9]. Microwave heating evenly enables reduced drying time and greater retention of bioactive compounds [10]. Hybrid techniques like microwave-assisted hot-air or vacuum drying further improve energy efficiency and increased drying rates without affecting the textural properties of onions [11,12]. Laguerre et al. [13] have worked on conventional hot-air as well as microwave drying of onions, indicating that the critical parameters affecting microwave drying are the microwave power, onion variety, and shape, whereas in conventional drying air temperature along with varieties will be critical.

Elsaeidy et al. [7] and Saeidy et al. [14] optimized the microwave drying parameters of onions based on drying kinetics and modeling. The evaluation of the application of mathematical models to analyze drying behavior and energy consumption [15] was found to depict a pattern independent of the factor of slice thickness, microwave power, and drying temperature [16,17]. This has allowed the application of sophisticated predictive modeling, including ANNs, to enhance energy efficiency in drying systems [16].

Traditional drying studies showed that onion slices dried by convection at 60 °C showed higher drying rates as the temperature rose, which was strongly influenced by the water content [18]. Infrared and microwave-assisted drying techniques markedly decreased drying durations.

The benefits of microwave drying, challenges still lie in final quality attributes, including rehydration properties, flavor retention, and color stability [19]. Recent innovations include microwave-assisted hydro-diffusion and gravity drying, which have shown great potential in successfully drying slices of onions [20,21]. Hybrid and advanced microwave-assisted techniques are under constant research to enhance drying efficiency and product quality further [[22], [23], [24]].

### Materials and methods

Sample Preparation: Onions were peeled, washed, and sliced into three different uniform thicknesses by a slicer that includes 7 mm, 9 mm, and 13 mm. The slices were pre-treated to remove the surface moisture, and they were stored under controlled conditions before drying.

**Fig. 1:**
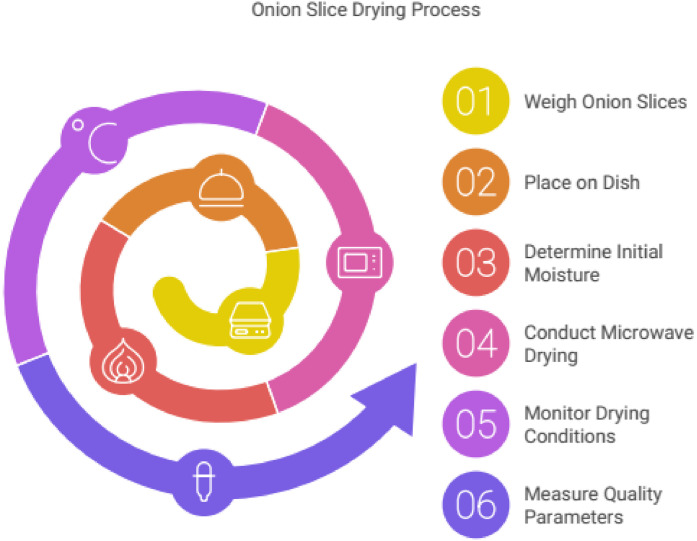
The model experimental procedure for microwave drying.

Drying Setup: Laboratory-grade microwave dryer with variable power levels from 200 W to 1000 W was used. The drying setup included real-time temperature monitoring with a thermal camera and digital thermometer to prevent overheating and ensure uniform drying. Each slice was placed in a perforated dish for adequate airflow. Determination of Moisture Content Oven drying was used as the reference method for determining moisture content. Samples were measured at regular intervals until moisture content stabilized for samples, the sample schematic experimental procedure has been presented in the Fig. 1.

Quality Evaluation: The dried samples were analyzed for color measurements using a colorimeter, texture analysis by a texture analyzer, and rehydration capacity. All these parameters help to know how the drying condition impacts the final product quality [4,16].

Mathematical Correlations: Formulations have been developed to predict the instantaneous moisture content in terms of drying rate, microwave power, and time. These correlations are checked with experimental data for accuracy and applicability [1].

This moisture content at an instant in time in a drying process can be estimated using the initial moisture content (M0), area (A), mass of solids (ms), time (t), average drying rate (Rd), and microwave heating power (P) in a correlation based on drying kinetics and energy balance. Here's a generalized form of such a correlation:

(1) $$M_{t}=M_{0}-\left(\frac{R_{d}At}{m_{s}}\right)\left(\frac{P}{P_{ref}}\right)$$

Where: (M_t_) is the instantaneous moisture content at time (t). (M_0_) is the initial moisture content. (A) is the surface area exposed to drying. (m_s_) is the mass of solids. (t) is the drying time. (R_d_) is the average drying rate. (P) is the microwave heating power. (P_ref_) is a reference power level for normalization.

This equation assumes the relationship between microwave power and rate of drying would be linear-which is a rather useful simplification for practical considerations; the actual correlation is likely to involve fitting with empirical data and, in any event, conditions associated with your actual drying process.

Mt represents the instantaneous moisture content of the material in microwave drying. This moisture content can be predicted through correlation based on heat and mass transfer principles. Usually, this is accompanied by process variables such as initial moisture content, area, mass of solids, time, average drying rate, and microwave heating power.

(2) $$M_{t}=M_{0}-R_{d}t\left(\frac{P}{Am_{s}}\right)$$

Where: M_t_: Instantaneous moisture content (kg water/kg dry solids) at time t; M_0_: Initial moisture content (kg water/kg dry solids); k: Drying rate constant (can be determined empirically, depending on material properties and drying conditions); t: Time of drying (s); P: Microwave heating power (W); A: Surface area available for drying (m²); m_s_: Mass of solids (kg).

### Method validation

**Fig. 2:**
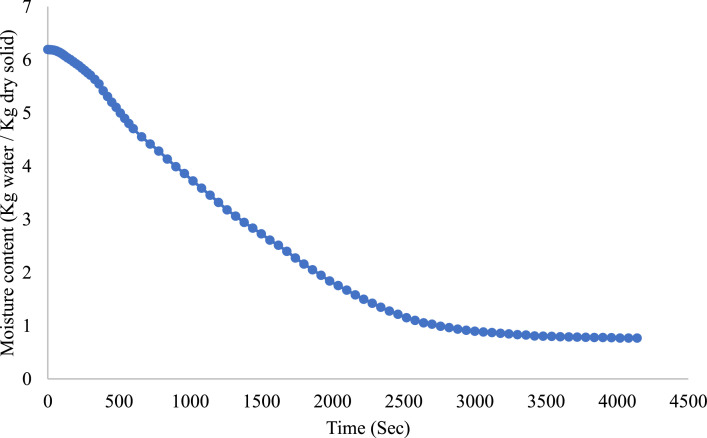
Moisture content variation with time for onions of size 7 mm thickness at 200 W for 7 mm onion thickness.

The drying behavior of onion slices in a microwave oven - moisture content variation with time has presented in Fig. 2. At the beginning, the moisture content of the onion slices is extremely high. In the drying time interval of the first 1000 s, the considerable decrease in moisture content points to a rapid drying stage. This is the free moisture evaporation stage in the phase of microwave heating. The rate of drying decreases significantly as the curve flattens, which indicates the falling rate stage. In this stage, the displacement of bound water takes place and diffusion of moisture becomes the limiting process. About 4000 s passed with the end result being the moisture content reaching a plateau of about 0.5 kg of water/kg dry solid, indicating attainment of equilibrium moisture. This is a characteristic phenomenon of desiccating biological materials, wherein their drying rate decreases with an increase in the strength of bound water within their structure.

### Impact of microwave power

**Fig. 3:**
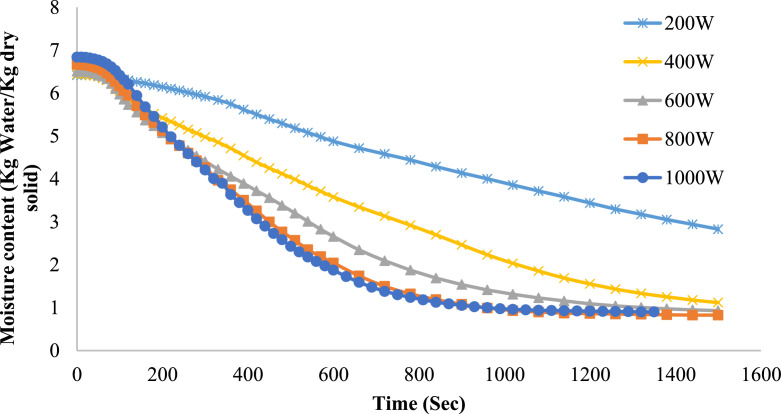
The moisture content variation with time at various Microwave heating powers for 7 mm thick onions.

Drying behavior of onion slices at varying microwave powers: 200 W, 400 W, 600 W, 800 W, and 1000 W is illustrated in Fig. 3. Moisture content (kg water/kg dry solid) vs time (seconds) The initial moisture content values are approximately uniform across all power situations, at about 7 kg of water per kg of dry product. The drying process progressively reduces moisture content over time, and elevated microwave power levels facilitate accelerated drying. At low power levels of 200 W and 400 W, the drying process is significantly prolonged, resulting in a gradual slope on the curve. Nevertheless, elevated power levels of 800 W and 1000 W signify a pronounced reduction in moisture content, suggesting expedited water drainage. Upon completion of the drying period, all power levels achieve identical final equilibrium moisture content; nonetheless, elevated power levels reach this state significantly more rapidly. This illustrates the direct impact of microwave power on drying efficiency, as increased power levels enhance moisture evaporation through greater energy input.

The drying curves indicated an initial quick phase of moisture removal, succeeded by a slower phase during which bound water was extracted. Elevated power levels (800 W and 1000 W) produced accelerated drying rates and reduced durations in comparison to lower ones (200 W and 400 W). These results align with earlier research indicating comparable developments in agricultural goods [6,11].

**Fig. 4:**
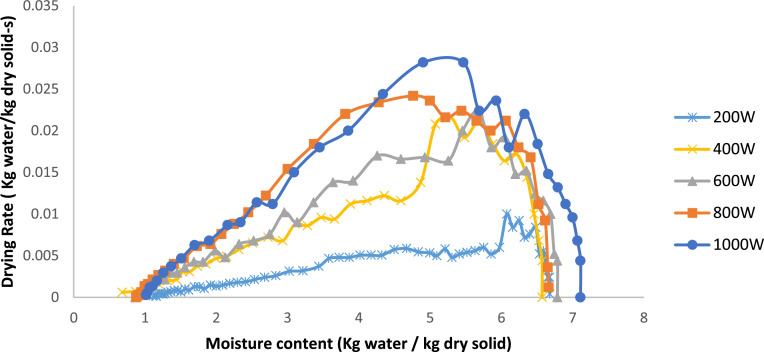
Drying rate variation with moisture content at various Microwave heating powers 7 mm.

Fig. 4 illustrates the drying rate (kg water/kg dry solid⋅s) of onion slices at different microwave power levels (200 W, 400 W, 600 W, 800 W, and 1000 W) in relation to moisture content (kg water/kg dry solid). The drying rate first increases with higher moisture content at all power settings, reaching a peak before decreasing as moisture content decreases.

Increased microwave power levels (e.g., 800 W and 1000 W) exhibit significantly enhanced drying rates, defined by distinct peaks indicating rapid moisture removal. Lower power levels (200 W and 400 W) have reduced peaks and a more gradual drying rate curve, signifying a slower drying process. This pattern highlights the influence of microwave power on drying kinetics; higher power levels provide more energy for water evaporation, leading to faster drying rates. As the moisture content approaches equilibrium, the drying rate decreases at all power levels due to the removal of bound water, requiring additional energy and time for evaporation. This pattern is indicative of microwave-assisted drying in agricultural commodities.

**Fig. 5:**
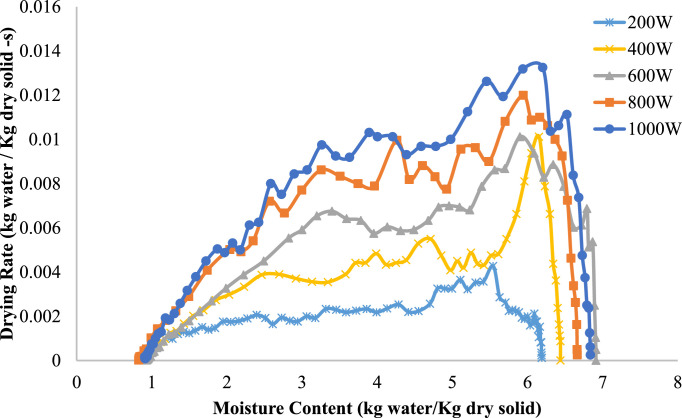
Drying rate variation with moisture content at various Microwave heating powers for 9 mm thick onions.

**Fig. 6:**
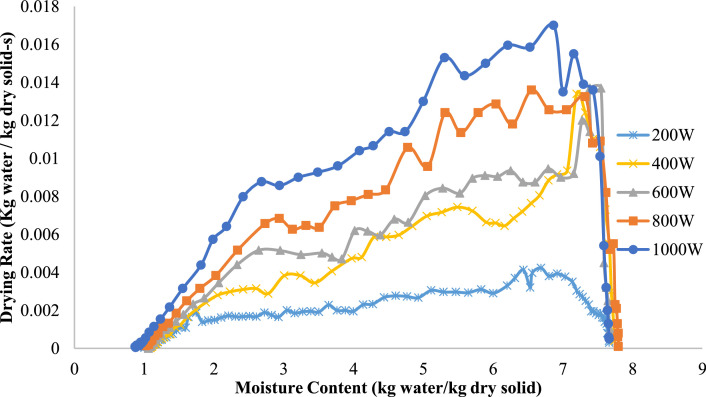
Drying rate variation with moisture content at various Microwave heating powers for 13 mm thick onions.

As a function of moisture content (kg water/kg dry solid), Fig. 5, Fig. 6 illustrate the drying rate (kg water/kg dry solid-s) of onion slices at various microwave power levels (200 W, 400 W, 600 W, 800 W, and 1000 W). The drying rate increases with moisture content at all power settings, reaching a peak before drastically decreasing as moisture content decreases. This pattern exemplifies the typical desiccation tendency of biological materials. At higher power levels (e.g., 800 W and 1000 W), the drying rates significantly increase, displaying sharper and more pronounced peaks compared to lower power levels (e.g., 200 W and 400 W). Increased power levels improve moisture extraction efficiency, resulting in shorter drying times. In contrast, with 200 W, the drying rate increases more slowly and consistently remains lower, indicating an extended drying duration. The notable decrease in drying rate with reduced moisture content is linked to the removal of bound water, which demands more energy and time than the extraction of free water. The graph demonstrates the substantial influence of microwave power on drying kinetics, where elevated power leads to quicker drying rates and improved efficiency. These findings highlight the imperative of modifying power input for effective drying processes in both commercial and laboratory environments.

### Predictive modeling

**Fig. 7:**
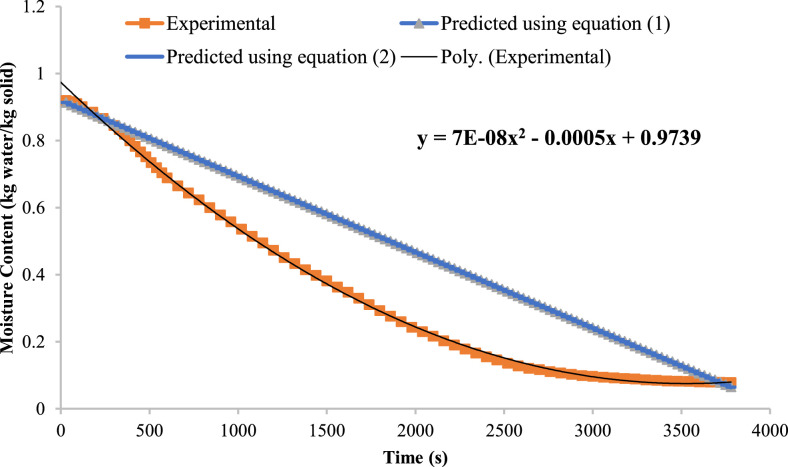
The instantaneous moisture content variation with time for different correlations.

Predicting the instantaneous moisture content is important to attain the right amount of moisture in the solids. Microwave drying shows a fast rate of drying as compared to conventional methods, and during such high rates, there is a high possibility of roasting or damaging the internal structure of particles. Therefore, it is essential to predict the instantaneous moisture. Here, two correlations have been developed taking into account several parameters such as initial moisture, average drying rate, microwave heating power, drying area, mass of solids, and time. The correlation developed is described in Eqs. (1) and (2). Fig. 7 gives the experimental and expected instantaneous moisture contents. This has led to the establishment of a second-order polynomial equation that is to be used in predicting the instant moisture content. The polynomial equation agrees with the experimental conditions, and therefore it is valid in accurately estimating the instant moisture content with time as illustrated by Feng et al. [1] and Kumar & Karim [24].

### Quality attributes

#### Color analysis

Studies into color and texture showed that a higher level of power retained the samples better than low levels and had less exposure to long drying. The ability to rehydrate was also another measure of the product's fitness for use; it was again higher at optimum levels of power. Similar results were obtained by [4,10] and [25].

**Fig. 8:**
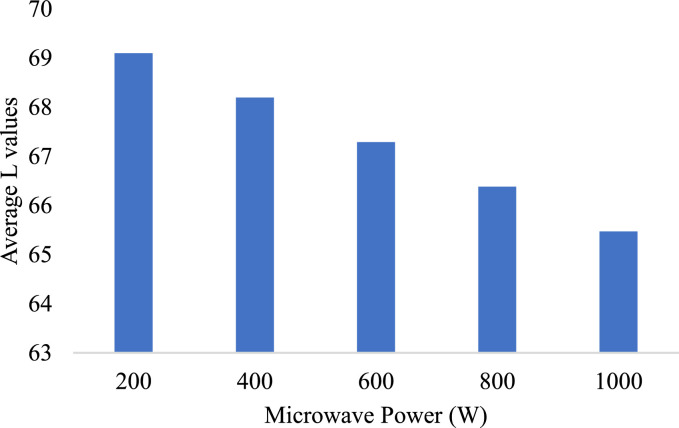
Average L values at various microwave Powers.

**Fig. 9:**
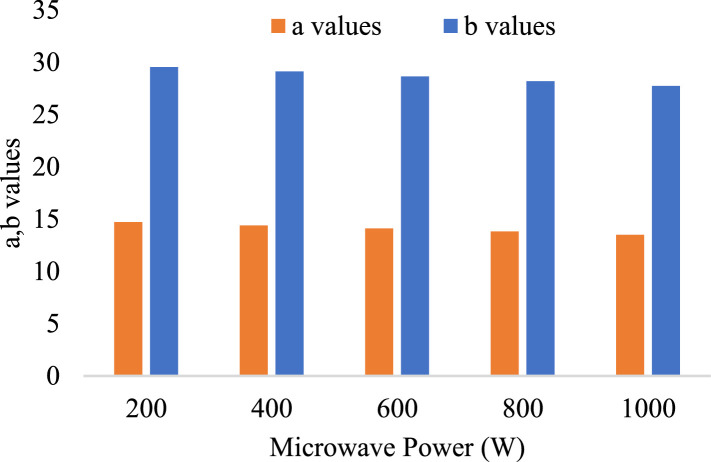
Average a,b values at various microwave Powers.

The bar graphs represented in Fig. 8 and Fig. 9 shows the comparative analysis of average L, a, and b values for different power levels. A very steep decrease in average l-value is observed with increased microwave power levels, indicating higher power produces greater overall darkening. A mild decrease is found in a, whereas a marked decrease in b is seen, which reflects the significant loss of yellow chromaticity at higher power levels. This indicates that the microwave power needs to be optimized so that color degradation does not occur with efficient drying.

In summary, high microwave power degrades color quicker and is vital to maintaining consumer acceptability within product quality requirements. These color visual trends tend to support an optimal balance in microwave drying with regard to quality retention versus energy efficiency.

### Texture analysis

**Fig. 10:**
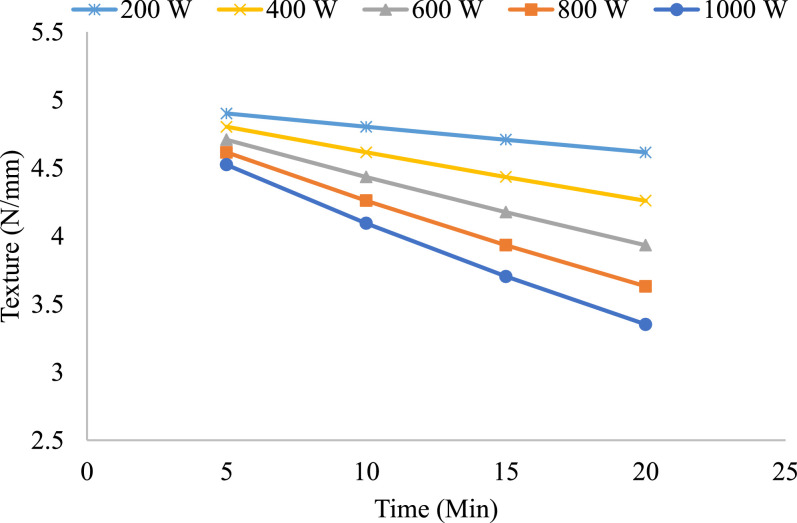
Onion slice texture variation at different time intervals of 7 mm.

**Fig. 11:**
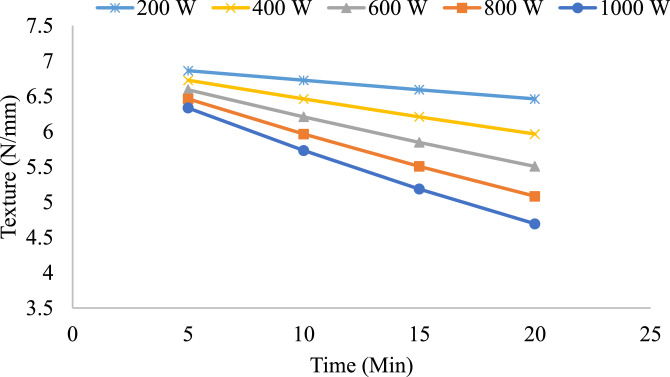
Onion slice texture variation at different time intervals of 9 mm.

**Fig. 12:**
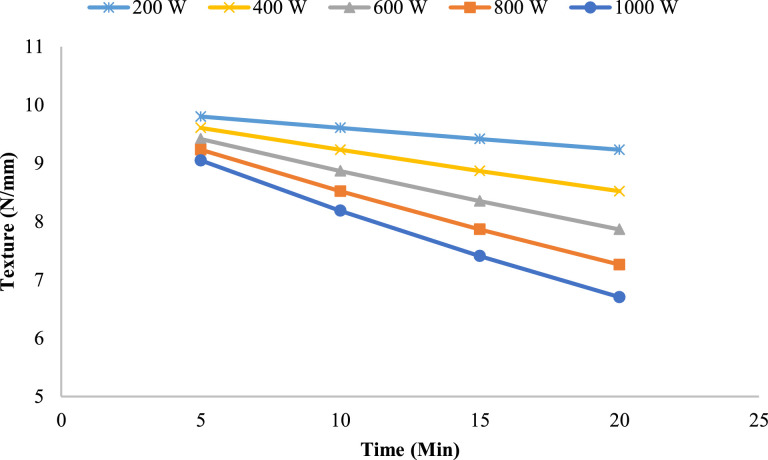
Onion slice texture variation at different time intervals of 13 mm.

The texture analysis of onion slices with thicknesses of 7 mm, 9 mm, and 13 mm, dried at microwave power levels of 200 W, 400 W, 600 W, 800 W, and 1000 W over 5, 10, 15, and 20 min, reveals significant trends in structural integrity during the drying process, the results are presented in the Fig. 10, Fig. 11, Fig. 12. The texture of thinner slices (7 mm) exhibited a pronounced decline over time, with higher power levels (800 W and 1000 W) causing rapid degradation. This can be attributed to the accelerated moisture removal and potential structural weakening at elevated energy inputs. At lower power levels (200 W and 400 W), the texture degradation was more gradual, suggesting better retention of structural integrity due to controlled drying rates. By 20 min, texture values for all power levels converged, indicating substantial softening regardless of the initial power.

Similar trends were also observed in the 9 mm slices with texture values decreased as a result of drying time. However, the increased thickness in the slices meant that texture values were still maintained at relatively higher levels as compared to 7 mm slices, especially at the lower power level. At the higher power levels, the breakdown was more critical after 10 min of drying, and once again, rapid energy input had proven detrimental to the structure. Nonetheless, thicker slices exhibited a slightly improved resistance to extensive drying than the thinner slices.

The highest initial texture values were found in the 13 mm slices, which had a greater structural mass. Lower power levels enabled these slices to maintain higher texture values for longer periods, with minimal degradation even at 10 min of drying. At higher power levels, the texture started falling sharply at around 10–15 min of drying time due to moisture loss and internal stresses resulting from non-uniform heating. For example, at the end of 20 min of drying time, the thicker slices maintained more structure compared to the thinner slices, thus showing more resistance to the severe drying conditions.

Overall, the results show that slice thickness, microwave power, and drying time play a critical role in texture preservation. Higher power levels dry the product faster but greatly compromise the structure, especially with thinner slices. Lower power levels provide a more controlled drying process, ideal for texture preservation. These results emphasize the need for optimized drying protocols that balance power input and drying duration to retain the desired texture in onion slices during microwave drying.

## Conclusion

The study has demonstrated that microwave drying is one of the best methods for minimizing drying time while preserving the quality of onion slices as compared to conventional drying methods. The results indicated that the higher microwave power levels (800 W and 1000 W) resulted in a faster drying rate and a decrease in moisture content within a relatively short period of time. This increase in energy input has a detrimental effect on some aspect of quality, mainly in terms of color and texture, which becomes increasingly so at higher power levels. Maintaining an appropriate balance between drying time, power levels, and slice thickness is thus important for product quality. This research, by developing mathematical correlations, gives insights into predicting moisture content during the drying process that can be useful in designing more efficient and sustainable microwave drying systems. Microwave drying has many advantages in terms of speed and energy efficiency, but future research needs to focus on the challenges in maintaining product quality, especially in optimizing power levels and drying duration for different slice thicknesses. More improvements in hybrid and microwave-assisted drying technologies would lead to efficiency in drying as well as lower degradation of the quality.

## Limitations

*Not applicable.*

## Ethics statements

*The authors declare that there are no conflicts of interest, and the research was conducted in accordance with ethical guidelines and approved protocols.*

## CRediT author statement

G Srinivas: Investigation and Formal analysis.

G Bhanu Radhika: Investigation and Resources.

BVS Praveen: Methodology and Conceptualization.

## AI tool usage statement

The paper does not used any AI tool in preparation of the manuscript.

## Declaration of competing interest

The authors declare that they have no known competing financial interests or personal relationships that could have appeared to influence the work reported in this paper.

## Data Availability

No data was used for the research described in the article.
